# Altered Expression Pattern of Molecular Factors Involved in Colonic Smooth Muscle Functions: An Immunohistochemical Study in Patients with Diverticular Disease

**DOI:** 10.1371/journal.pone.0057023

**Published:** 2013-02-20

**Authors:** Letizia Mattii, Chiara Ippolito, Cristina Segnani, Barbara Battolla, Rocchina Colucci, Amelio Dolfi, Gabrio Bassotti, Corrado Blandizzi, Nunzia Bernardini

**Affiliations:** 1 Unit of Histology and Medical Embriology,Department of Clinical and Experimental Medicine, University of Pisa, Pisa, Italy; 2 Unit of Pharmacology, Department of Clinical and Experimental Medicine, University of Pisa, Pisa, Italy; 3 Unit of Gastroenterology and Hepatology, Department of Clinical and Experimental Medicine, University of Perugia, Perugia, Italy; University of Navarra School of Medicine and Center for Applied Medical Research (CIMA), Spain

## Abstract

**Background:**

The pathogenesis of diverticular disease (DD) is thought to result from complex interactions among dietary habits, genetic factors and coexistence of other bowel abnormalities. These conditions lead to alterations in colonic pressure and motility, facilitating the formation of diverticula. Although electrophysiological studies on smooth muscle cells (SMCs) have investigated colonic motor dysfunctions, scarce attention has been paid to their molecular abnormalities, and data on SMCs in DD are lacking. Accordingly, the main purpose of this study was to evaluate the expression patterns of molecular factors involved in the contractile functions of SMCs in the *tunica muscularis* of colonic specimens from patients with DD.

**Methods and Findings:**

By means of immunohistochemistry and image analysis, we examined the expression of Cx26 and Cx43, which are prominent components of gap junctions in human colonic SMCs, as well as pS368-Cx43, PKCps, RhoA and αSMA, all known to regulate the functions of gap junctions and the contractile activity of SMCs.

The immunohistochemical analysis revealed significant abnormalities in DD samples, concerning both the expression and distribution patterns of most of the investigated molecular factors.

**Conclusion:**

This study demonstrates, for the first time, that an altered pattern of factors involved in SMC contractility is present at level of the *tunica muscularis* of DD patients. Moreover, considering that our analysis was conducted on colonic tissues not directly affected by diverticular lesions or inflammatory reactions, it is conceivable that these molecular alterations may precede and predispose to the formation of diverticula, rather than being mere consequences of the disease.

## Introduction

Diverticular disease (DD) is one of the most common pathologic conditions affecting the gastrointestinal tract in Western countries. Its pathogenesis is likely multifactorial in nature, involving dietary habits as well as changes in colonic pressure, motility and wall structure associated with ageing [1]. Abnormalities of colonic motility, including increments of the overall motor activity, as well as abnormal responses to physiologic stimuli and retro-propagation of mass movements, might predispose to the formation of pulsion diverticula by herniation of colonic wall [2]–[4].

Intestinal motility is regulated by complex interactions among smooth muscle cells (SMCs) of the gut *tunica muscularis*, enteric nerves and interstitial cells of Cajal (ICCs). Several lines of evidence from DD patients suggest that the diverticular colon displays substantial structural alterations of the enteric nervous system, mainly charaterized by myenteric and submucosal oligo-neuronal hypoganglionosis [5], a predominance of cholinergic nerves [6], and a significant decrease in myenteric glial cells and neuromuscular ICCs [7]. Other studies, performed *in vitro* on DD colonic samples, have shown an impairment of the tachykininergic pathway. In particular, colonic preparations, containing the circular (CM) and longitudinal muscle (LM) with the embedded myenteric plexus and the serosal layer, displayed a decreased contractile response to exogenous substance P in combination with a down-regulation of tachykininergic receptor genes [8]–[9]. Studies on colonic smooth muscle tissue from patients with DD have also demonstrated a marked thickening of the CM layer, in concomitance with shortening of the *taeniae*, which appears to be secondary to an abnormal elastin deposition [3]. However, studies focused on the molecular features of SMCs in the *tunica muscularis* of DD human colon are lacking.

Despite colonic SMCs are not all individually innervated, they are able to generate coordinated patterns of motor activity, acting as a functional syncithium. Indeed, the large number of gap junctions, which are abundantly present along SMC plasma membranes, allow the neurogenic electrical stimuli to spread from activated neighbouring cells. Gap junctions are clusters of intercellular channels, which consist of two apposing connexon complexes. Each connexon comprises six connexin (Cx) proteins, which allow the direct diffusion of ions and small molecules between adjacent cells [10]. Among the Cxs, Cx43 is the most widely expressed as well as the most represented in SMC gap junctions, including those of the human gut [11]. The functions of gap junctions can be regulated at different levels by a variety of mechanisms such as: modulation of connexon densities on cell membranes; Cx phosphorylation, which leads to modification of channel conductance as well as Cx trafficking and degradation [12]; protein kinase C (PKC) and RhoA activation [13]–[14]. As far as the family of Rho proteins is concerned, these are known to act as key regulators of several processes associated with changes in actin cytoskeleton, such as cell migration, adhesion and contraction [15]–[16]. In particular, RhoA/ROCK and PKC/CPI-17 represent the major molecular pathways, known to be involved in the late phase of smooth muscle contractility, including that of gastrointestinal SMCs [17].

Based on the above considerations, the present study was aimed at performing immunohistochemical studies on the distribution and quantitative expression of Cx isoforms, Cx43 phosphorylated at serine 368 (pS368-Cx43), PKC phosphorylated substrates, and RhoA signaling in the neuromuscular compartment of colonic samples from DD patients, by comparison with normal specimens. In addition, the ICC distribution and density within the *tunica muscularis* were studied by means of a morphometric method. Our findings revealed significant abnormalities in DD samples, concerning both the expression and distribution patterns of most of the investigated molecular factors and ICC.

## Materials and Methods

### Ethics statement

Care was taken to perform the study in full accordance with local ethical guidelines and recommendations by the Declaration of Helsinki (Seoul revision, 2008). However, since this was a retrospective study on archival material, no individual patient identification was involved and no study-driven clinical intervention was performed, no approval by ethical committee and no informed consent were necessary. The original patients gave their consent to have their tissue samples archived and used for scientific purposes. All patients' data were strictly de-identified and analyzed anonymously.

### Patients and tissue samples

The study was carried out on full-thickness archival samples of left (descending and sigmoid) colon collected from 10 patients (6 women, 4 men; age range 33–82 years), who underwent elective abdominal surgery for DD, after the third or fourth attack of diverticulitis [18]–[19]. Care was taken to select tissue samples, which included *taeniae* and displayed a microscopically normal aspect, without leukocyte infiltrate, while samples showing leukocyte infiltrate were avoided. Archival colonic samples from 8 subjects (3 women, 5 men; age range 42–80 years), who had undergone elective left hemicolectomy for non obstructing colon cancer, were used as controls. These samples, also selected from areas including *taeniae*, were tumour-free and chosen at a distance of at least 5 cm from the resection margin samples. The routinely fixed and processed colonic samples were serially cross-sectioned to obtain 8 µm-thick sections with SMCs of the circular layer and myenteric ganglia cut longitudinally. Immediately before use, slides were deparaffinized, rehydrated and processed for both routine haematoxylin-eosin staining and immunohistochemistry. For each selected colonic sample, at least 3 serial sections (1/18) were examined.

### Immunohistochemistry

The slides were exposed to microwaves for 15 min at 600 W in 10 mM sodium citrate for antigen retrieval, incubated with 3% H_2_O_2_/dH_2_O for 15 min, and with normal goat serum (1∶20, Vector, Burlingame; CA, USA) for blocking endogenous peroxidase and non-specific binding, respectively; thereafter, they were incubated with primary antibodies in 0.1% BSA-PBS overnight at 4°C. Optimal working dilutions of primary antibodies, determined empirically by means of serial dilutions, are summarized in Table 1.

**Table 1:** Primary antibodies used in this study.

Antibody	Species	Source	Dilution
Anti-human Cx43	Mouse monoclonal	Santa Cruz Biotechnology, Santa Cruz, CA, USA	1∶4500
Anti-human phospho-Cx43 (pS368-Cx43)	Rabbit polyclonal	Cell Signaling Technology, Beverly, MA, USA	1∶50
Anti-human Cx26	Rabbit polyclonal	Santa Cruz Biotechnology	1∶200
Anti-human RhoA	Rabbit polyclonal	Santa Cruz Biotechnology	1∶2500
Anti-phospho-PKC substrate (PKCps)	Rabbit polyclonal	Cell Signaling Technology	1∶800
Anti-c-kit (CD117)	Rabbit polyclonal	DakoCytomation,Glostrup, Denmark	1∶200
Anti-smooth muscle α-actin (1A4)	Mouse monoclonal	Cell Marque, Rocklin, CA, USA	undiluted

Detection was carried out by sequential treatments with biotinylated anti-mouse/rabbit immunoglobulins (1∶200, Vector), strepavidin-peroxidase complex (Vector), 3.3′-diaminobenzidine tetrahydro-cloride (DAB, DakoCytomation) or DAB enhanced with 10% nickel chloride (Sigma-Aldrich, St. Louis, Mo, USA) for ICC detection. Sections were counterstained with Harris's haematoxylin or nuclear fast red (Fluka, Buchs, Switzerland) for anti-c-Kit immunostaining. Detection of smooth muscle α-actin (α-SMA) was performed using the Cell Marque detection kit (Cell Marque, Rocklin, CA, USA) in accordance with the manufacturer's instructions. Negative controls were obtained by omitting primary antibodies or substituting them with pre-immune mouse/rabbit serum (1∶100).

**Figure 1:**
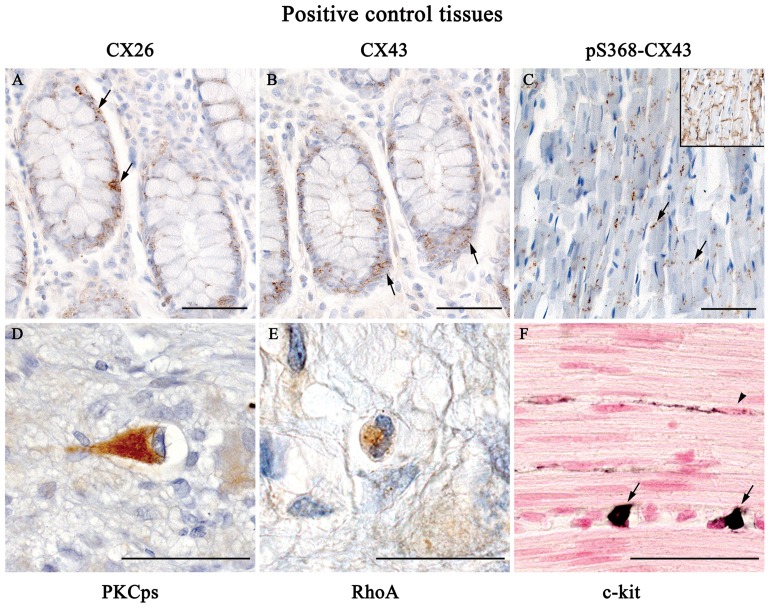
Positive control tissues for immunohistochemistry. (A,B) The crypts of normal human colon were selected as control positive structures for Cx26 (A) and Cx43 (B): immunoreactivity is evident in the ephitelial cells of crypts (arrows). (C) Human myocardium was used as positive control for pS368-Cx43: immunoreactivity is localized at level of intercalated disks (arrows); immunoreactivity of myocardium for Cx43 is displayed in the box. (D) A human myenteric neuron, taken as a positive control cell for PKC, displays an intense PKCps immunoreaction. (E) A granulocyte of normal colon, chosen as positive control for RhoA, is markedly immunoreactive. (F) Mast cells (arrows), locked in the *tunica muscularis* of human normal colon, show a strong immunoreaction for c-Kit. In the same field an intramuscular c-Kit positive ICC is also evident (arrowhead). Scale bar: 50 µm.

To test the antibody specificity the following positive control tissues were used as shown in Fig. 1: human colonic crypt epithelium for Cx26 and Cx43 [11]; human myocardium for pS368-Cx43 [12], [20]; human enteric neurons for protein kinase C phosphorylated substrates (PKCps) [21]–[22]; human granulocytes for RhoA [23]; human mast cells for c-Kit [24]. All reactions were performed in humid chambers at room temperature, unless otherwise specified. Sections were then examined by means of a Leica DMRB light microscope and DFC480 digital camera (Leica Microsystem, Cambridge, UK) to capture representative photomicrographs and microscopic fields for quantitative evaluations.

### Quantitative analysis of immunolabelled Cx26, Cx43, RhoA and PKCps

Three sections were examined for each colonic sample. For each section, 10 randomly selected microscopic fields, focused on the *tunica muscularis*, were captured at 400× magnification and analyzed by Image Analysis System (McBiophotonics Image J) as previously reported [24]. Briefly, 5 fields were focused on CM and 5 on LM. Each field was subjected to intensity thresholding, depending on signal-to-noise ratio in order to highlight the immunopositive area, which was expressed as percentage of the total tissue area examined (percentage positive pixels [PPP]). For each sample, the percentages of immunoreactive areas were expressed as means ± SD of PPP estimated on 10 fields examined for 3 sections. For each group (controls and DD patients), the value of immunopositive area was expressed as mean ± SD of PPP estimated in controls or patients.

### Quantitative analysis of ICCs

Only c-Kit-immunostained cells with red nuclei were considered for ICC counting and density estimation, which was performed as previously reported, with exclusion of c-Kit-positive cells displaying the morphology and size of mast cells [24]. Briefly, for each section, 20 randomly selected microscopic fields were captured at 200× magnification. Ten fields were focused on CM, and 10 on LM to count ICC-CM, and ICC-LM, respectively. ICC counting was performed by a squared grid and the density for each patient was expressed as ΣICC number/Σtissue area (mm^2^) found in the 3 serial sections (20 fields/section). For each patient at least 20 mm^2^ (10 mm^2^ for CM and 10 for LM) were examined. For each group (controls and DD patients), the density of ICC was expressed as mean ± SD of ICC density estimated in controls or patients.

### Statistical analysis

Statistical analysis was carried out using the Statistical Package for Social Sciences software (SPSS version 17.0). Mann-Whitney *U*-test for unpaired data was used to assess the statistical differences between groups; *p* values <0.05 were considered to be significant.

## Results

Histopathologic analysis, carried out by conventional haematoxylin and eosin staining, revealed a normal morphology, without leukocyte infiltrate, in all sections of colonic samples from control subjects and DD patients. However, some specimens from DD patients showed a trend toward an increased thickness of the smooth muscle layer. All markers under study were found to be expressed in the respective positive control tissues (Fig. 1), and they were not detected in negative controls.

### Expression of Cxs

**Figure 2:**
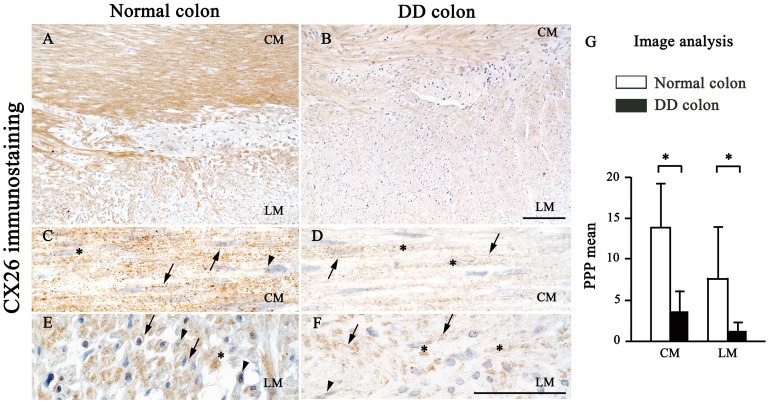
Cx26 immunostaining and quantitative analysis in the neuromuscular compartment of normal and diverticular disease human colon. In normal colon, Cx26 is widely expressed in both circular (CM) and longitudinal muscle (LM) (A), while it is decreased in samples from DD colon (B); scale bar: 100 µm. At higher magnification (C–F), SMCs show a punctuated immunoreactivity, which is localized at the membrane (arrows), cytoplasmic (asterisks), paranuclear and nuclear (arrowheads) levels; scale bars: 50 µm. (G) Image analysis of Cx26 expression. Each column represents the PPP mean ± SD (9<n<11). *P<.05, significant difference vs normal colon.

**Figure 3:**
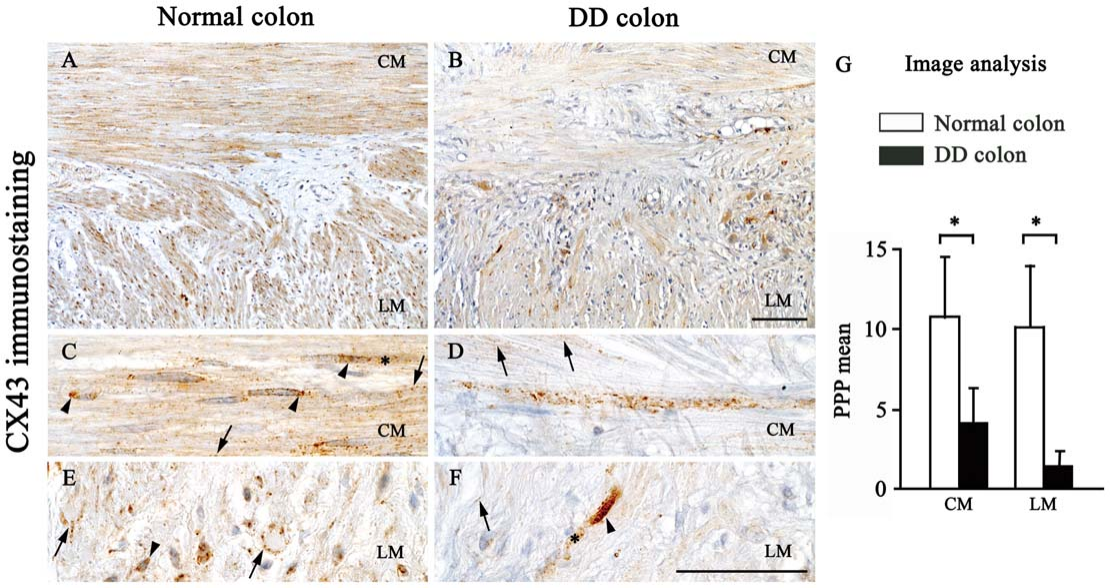
Cx43 immunostaining and quantitative analysis in the neuromuscular compartment of normal and diverticular disease human colon. In normal colon, appreciable amounts of Cx43 are expressed in the circular (CM) and longitudinal muscle (LM) (A), while Cx43 expression is decreased in samples from DD colon (B); scale bar: 100 µm. At higher magnification (C–F), SMCs show a punctuated immunoreactivity, which is localized at the membrane (arrows), cytoplasmic (asterisks), paranuclear and nuclear (arrowheads) level; scale bars: 50 µm. (G) Image analysis of Cx43 expression. Each column represents the PPP mean ± SD (8<n<10). *P<.05, significant difference vs normal colon.

Appreciable amounts of Cx26 and Cx43 immunostaining were detected in SMCs of the *tunica muscularis* of all colonic specimens. Normal colonic samples displayed a marked staining for both Cx isoforms in the CM and LM layers, although the immunoreaction products were mainly appreciable at level of the CM (Fig. 2A and Fig. 3A). A spotted immunoreactivity was evident by high magnification within SMCs, being mostly localized at the membrane level. Specific immunostaining was also appreciable at cytoplasmic and, for Cx43 in particular, at paranuclear and nuclear level in a variety of cells (Fig. 2C,E and Fig. 3C,E).

In DD colonic samples, these patterns were markedly altered: Cx26 and Cx43 positivity were reduced or focally lost in both CM and LM layers of all pathological samples (Fig. 2B and Fig. 3B). With regard for the pattern of cellular Cx distribution, there were not remarkable variations, with exception for the paranuclear localization, which appeared to be very faint (Fig. 2D,F and Fig. 3D,F). This reduction was confirmed by quantitative analysis, which highlighted a significantly lower percentage of Cx immunostained areas in colonic DD samples, as compared to controls (Fig. 2G, Fig. 3G).

**Figure 4:**
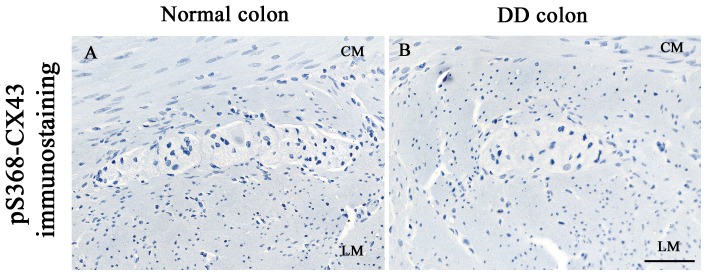
Cx43 phosphorylated at serine 368 (pS368-Cx43) immunostaining. No immunoreactivity is evident in the *tunica muscularis* of normal (A) and DD (B) human colon; scale bar: 100 µm. (C–D).

No appreciable immunoreactivity for pS368-Cx43 was detected in SMC of the *tunica muscularis* in both controls and DD samples (Fig. 4). Nevertheless, the validity of immunostaining procedure was testified by the marked pS368-Cx43 positivity detected at level of intercalated disks of the myocardium, which is regarded as a reliable positive control tissue for this antigen [13], [20] (Fig. 1).

### Expression of molecular factors involved in the contractile function of SMCs

**Figure 5:**
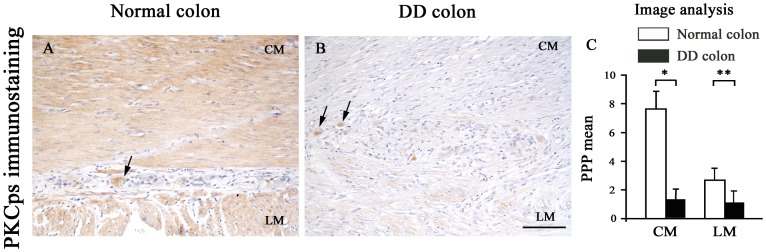
PKC phosphorylated substrates immunostaining and quantitative analysis in the neuromuscular compartment of normal and diverticular disease human colon. In normal colon, PKCps immunostaining is expressed in both circular (CM) and longitudinal muscle (LM) (A), while it is reduced in DD (B); scale bars: 100 µm. Myenteric neurons (arrows) are intensely immunostained. (C) Image analysis of PKCps expression. Each column represents the PPP mean ± SD (9<n<11). **P<.005 and *P<.05, significant differences vs normal colon.

The activity of PKC was studied by detecting the levels of phosphorylated PKC substrates. A diffuse, marked PKCps immunostaining was observed in SMCs of all normal colonic samples at level of the *tunica muscularis*. By contrast, a significant decrease in PCKps immunostaining was found in DD samples (Fig. 5A,B), as confirmed by quantitative analysis (Fig. 5C).

**Figure 6:**
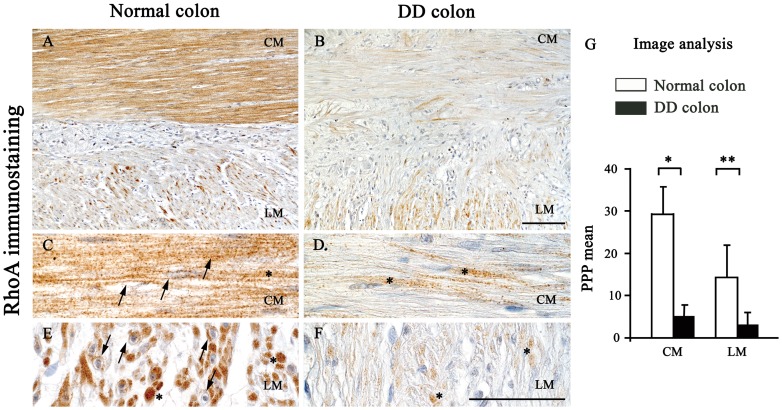
RhoA immunostaining and quantitative expression in the neuromuscular compartment of normal and diverticular disease human colon. In normal colon, RhoA is expressed in the circular (CM) and longitudinal muscle (LM) (A), while it is reduced in DD (B); scale bar: 100 µm. At higher magnification, SMCs show a dotted immunoreactivity localized at the membrane (arrows) and cytoplasmic (asterisks) level in normal colon (C,E), while in DD samples the localization is mainly evident in the cytoplasm (asterisks) (D,F); scale bars: 50 µm. (G) Image analysis of RhoA expression. Each column represents the PPP mean ± SD (9<n<11). **P<.005 and *P<.05, significant differences vs normal colon.

With regard for RhoA, two patterns of immunostaining could be appreciated: 1) a cytoplasmic staining, corresponding to inactivated GDP-bound RhoA, and 2) a plasma membrane staining, corresponding to activated GTP-bound RhoA [23], [25]–[26]. A dotted, strong RhoA immunolabelling, distributed at level of both cytoplasm and plasma membrane, was evident in SMCs of the *tunica muscularis* in normal colon (Fig. 6A,C,E). By contrast, the SMCs of DD colonic samples displayed a weak RhoA immunoreactivity, which was mostly localized in the cytoplasm (Fig. 6B,D,F). Quantitative analysis confirmed the decreased RhoA expression in colonic DD sections, as shown by the significant differences between the PPP means of these two groups (Fig. 6G).

**Figure 7:**
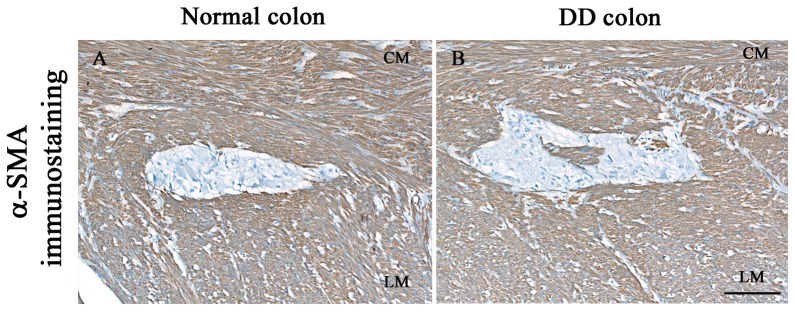
Smooth muscle α-actin (α-SMA) immunostaining. α-SMA is equally expressed in circular (CM) and longitudinal muscle (LM) of normal (A) and diverticular disease (DD) (B) human colon. Scale bar: 100 µm.

α-SMA immunoreactivity labelled all the components of smooth muscle layers including the SMCs of *tunica muscularis*. No appreciable differences in the distribution patterns or the quantitative expression of α-SMA were observed in the neuromuscular compartment when comparing DD colonic specimens with normal colon (Fig. 7).

### c-Kit positive ICCs

**Figure 8:**
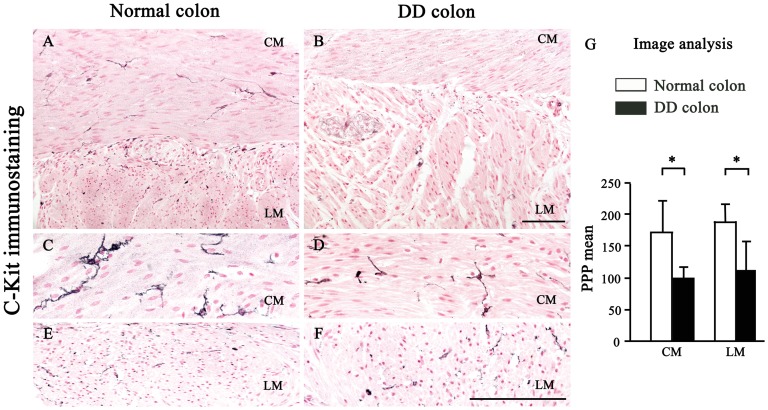
Immunostaining and quantitative analysis of ICCs in neuromuscular compartment of normal and diverticular disease human colon. Colonic specimens from DD patients contain fewer c-Kit positive and nuclear fast red-counterstained ICCs, with blunted and shortened branching (B, D, F), as compared to normal colon. Scale bars: 100 µm (A, B) and 50 µm (C–F). (G) Column graphs display ICC counting which is given as means ± SD of individual ICC densities normalized to area for ICC-CM (C) and ICC-LM (I).*P<.05,significant difference vs normal colon.

ICCs were identified by black c-Kit immunostaining and red nuclei (Fig. 8). Strongly c-Kit immunostained cells, with morphological appearance and size of mast cells, were excluded from the analysis and were taken as internal positive controls (Fig. 1). Normal colonic samples from control subjects displayed an abundant presence of c-Kit-positive dendritic cells, widely spread throughout the *tunica muscularis*, with their long processes running along the major axis of SMCs. As compared to normal controls, colonic specimens from DD patients showed considerable alterations of the intramuscular ICC appearance and distribution pattern. In particular, the c-Kit network was rarefied and disarranged as a result of: 1) significant morphological alterations of ICCs, which showed curtailed and blunted processes (Fig. 8B,D,F); 2) decrease in ICC density in both CM and LM (Fig. 8G).

## Discussion

Intestinal motility is ensured by coordinated contractions of the *tunica muscularis*, which depend from finely tuned interactions among the myenteric motor neurons, enteric glial cells, ICCs and SMCs of the CM and LM layers. Not all the intestinal SMCs are individually innervated by enteric nerves and, for this reason, their plasma membranes are equipped with a number of gap junctions, built up by a complex coaxial arrangement of transmembrane channels. These intercellular junctions allow the transmission of signaling molecules from innervated SMCs to non-innervated neighboring SMCs, thus orchestrating a coordinated motor activity and the implementation of different bowel motor programs. Among the molecular mechanisms which control the morphology and functions of SMC gap junctions, it is being increasingly appreciated that their permeability is regulated by both RhoA monomeric GTPase, an emerging key regulator of SMC phenotype [14], and PKC [13], [27]. Interestingly, RhoA and PKC are also involved in the control of SMC contraction [17].

To date, histopatological studies conducted on gastrointestinal neuromuscular disorders have been mainly focused on the enteric nervous system and ICCs, which have been found to be both variously affected under different pathological conditions. By contrast, although SMCs are well recognized as the final effectors of enteric neuromuscular units and extensive electrophysiological studies on SMCs have investigated the area of colonic motor dysfunctions [28], scarce attention has been paid to the molecular abnormalities of enteric musculature in gut dysmotility [29]–[32], and data on SMCs in DD are lacking. Thus, we evaluated the expression patterns of molecular factors involved in the contractile functions of SMCs in colonic specimens from patients with DD. In particular, we examined the expression of Cx26 and Cx43, which are prominent components of gap junctions in human colonic SMCs [11], and an array of signaling factors, which are known to regulate both the functions of gap junctions and the contractile activity of SMCs. Furthermore, due to the important role that ICCs play in the electrical activity of SMCs, we deemed interesting to evaluate also the distribution and density of ICCs in the *tunica muscularis* of DD patients.

In the present study, all normal and DD colonic specimens, examined by haematoxylin-eosin staining, displayed normal morphological patterns, without significant evident histopathological abnormalities in the neuromuscular compartment. Despite this finding, the immunohistochemical analysis revealed significant abnormalities in DD samples, concerning both the expression and distribution patterns of most of the investigated molecular factors. Indeed, with exclusion of α-SMA and pS368-Cx43, whose expressions were found to be similar in DD and normal samples, all the remaining factors (Cx26, Cx43, PKCps and RhoA) were markedly reduced.

Immunolabelling for Cx26 and Cx43 was positive, with different degrees of expression, at level of the *tunica muscularis* in the SMCs of normal and DD colonic specimens, both in CM and LM layers. Of note, these Cx isoforms were not detected in LM of the normal human colon in previous investigations [11]. However, different immunostaining conditions might account for this discrepancy. Indeed, when our experiments were repeated in accordance with the procedure described by Kanczuga-Koda et al. [11], Cx26 and Cx43 immunolabelling could not be visualized in the LM of cross-sectioned colonic samples (data not shown). This circumstance emphasizes the concept that comparable morphological examinations can be obtained only by a very close adherence to standardized and validated procedures, which have been largely advocated, particularly in the field of gastrointestinal neuromuscular diseases [33]–[35].

Based on the role traditionally ascribed to gap junction channels, the dramatic reduction of Cx expression found in DD samples, at all cellular levels, could represent a correlate of impaired junctional communications among adjacent SMCs. However, besides the role played by Cxs in setting up gap junctions, recent studies have demonstrated that hemichannels, expressed at level of unopposed SMC membranes, can function independently of cell-to-cell communication and can mediate the exchange of small molecules between cells and the extracellular environment [36]. In addition, membrane Cx43 regulates intracellular signaling, acting as a scaffold which fosters protein–protein interactions through the domains located in its cytoplasmic C-terminus tail [36]–[38]. Furthermore, it has been shown that intracellular Cxs may play a role as signal transduction molecules involved in the modulation of cell proliferation and tumour progression, as previously observed in human cancer cell lines [39], as well as in human gastrointestinal tumours [40]–[41]. Thus, in keeping with current knowledge, the membrane localization of Cx proteins, as detected in our study, might reflect their presence in the structure of gap junctions or their function as scaffold proteins involved in signal transduction. On the other hand, the cytoplasmic and paranuclear localizations of Cxs might be associated with molecule trafficking towards or from SMC plasma membranes, during the process of setting up or disassembling gap junctions, as well as with signal proteins translocating to the nucleus. In this respect, the decrease in Cx expression, as observed in our DD samples, could explain the concomitant decrease in RhoA and PKCps expression, since Cxs might be also involved in the control of these signaling pathways.

Previous evidence supports the view that Cx phosphorylation has relevant modulating actions on gap junction activity. Therefore, we examined the expression of pS368-Cx43, which has been recently demonstrated to reduce the conductance of gap junction channels in both myocardiocytes [13] and vascular SMCs [42]. Since in all our specimens, from either normal or DD colon, the expression of pS368-Cx43 could not be detected, despite its presence in positive control tissues, it can be argued that this molecular mechanism may not be involved in the control of gap junction permeability at level of human colonic SMCs. However, other molecular sites of Cx43 phosphorylation, differing from Ser368, or other phosphorylated Cx isoforms, might be involved in the regulation of channel permeability, and these possibilities deserve careful consideration in future studies.

With regard for the molecular factors known to regulate the permeability of Cx43-based channels, the activation of PKC and RhoA has been considered in the present study. Indeed, as anticipated above, PKC can induce the reduction of gap junction conductance by direct phosphorylation of Cx43 at serine 368 in myocardial tissue [13]. By contrast, RhoA activation has been reported to enhance the processes of cell-to-cell solute diffusion, without modifying the cellular redistribution of junctional plaques or the Cx43 phosphorylation pattern in myocardiocyte plasma membranes [14]. Furthermore, RhoA/ROCK and PKC/CPI-17 have been found to represent the relevant molecular pathways controlling the contractility of gastrointestinal SMCs. In particular, PKC and RhoA signaling can positively influence the late phase of contraction, acting by two parallel signal pathways, which ultimately lead to the inhibition of myosin light chain phosphatase, with a consequent enhancement of tonic SMC contractile activity. It is also interesting to note that different roles can be played by RhoA and PKC pathways depending on the anatomical region at which the motor activity is considered [17]. When referring our results to this background knowledge, the significant decrease in RhoA expression, and activity in DD colonic SMCs, as suggested by its reduced plasma membrane content, could reflect an impairment of intercellular communications among SMCs. By contrast, when considering the decrease in PKC activity, as demonstrated by the reduction of PKC phosphorylated substrate expression, this pathway does not appear to be involved in the control of intercellular communication by Cx43 phosphorylation at Ser368. This view is supported by our observation showing that the appreciable amount of PKC substrates, detected in normal colon, were not associated with the expression pattern of immunolabelled pS368-Cx43. As an alternative explanation, the reduced RhoA and PKCps immunostaining in DD colon might be representative of an altered condition of intracellular signaling regulating SMC contraction. In particular, the altered phenotype observed in SMCs of DD samples could be compatible with an inhibition of the RhoA/ROCK pathway. Interestingly, such a condition, which might lead to loosening of colonic *tunica muscularis* in DD patients, has been shown to directly affect the relaxation of SMCs [17]. Overall, in predisposed individuals these changes might facilitate a collapse and/or weakening of tensile strength in the *tunica muscularis*, with a consequent risk of forming diverticula by herniation of the colonic wall, especially in the sigmoid colon, where the pressures toward the wall are greater, and even under normal physiological conditions there is a continuous motor activity acting as a physiological brake on the colonic content [43]–[44].

Within a framework of SMC failure, characterized by a significant decrease in the expression/activity of molecular factors involved in the control of SMC contractility, the role played by ICCs in the modulation of enteric neuromuscular transmission deserves a careful consideration. These cells function primarily as pacemakers, but they can also act as cellular bridges connecting nerve varicosities with several SMCs, thus ensuring a proper diffusion of contractile inputs throughout the *tunica muscularis* [45]. In this respect, the analysis of neuromuscular ICC density, as performed in the present study, highlighted a significant decrease in the density of these cells throughout the whole *tunica muscularis* of DD patients, at level of both CM and LM layers, in agreement with previous results reported by Bassotti et al. [7]. Of note, patients with DD have been shown to display an increase in both overall and rhythmic colonic contractions [2], [46]. Since studies on rat [47] and human [48] colonic muscle have suggested that ICCs are likely to inhibit colonic motility by nitric oxide release, the reduction of intramuscular ICC, as observed in the colon of DD patients, might result in an increment of SMC contractility, as repeteadly observed in these patients [4].

In conclusion, the present results provide the first evidence of altered expression patterns of important molecular factors involved in the regulation of SMC contractility at level of the *tunica muscularis* of DD patients. Considering that our analysis was conducted on colonic tissues, which were taken from DD patients but were not directly affected by diverticular lesions or inflammatory reactions, it is conceivable that these molecular alterations, as highlighted by the present immunohistochemical assay, may precede and predispose to the formation of diverticula, rather than being mere consequences of the disease.
